# Systematic identification of Ctr9 regulome in ERα-positive breast cancer

**DOI:** 10.1186/s12864-016-3248-3

**Published:** 2016-11-09

**Authors:** Hao Zeng, Li Lu, Ngai Ting Chan, Mark Horswill, Paul Ahlquist, Xuehua Zhong, Wei Xu

**Affiliations:** 1 McArdle Laboratory for Cancer Research, Wisconsin Institute for Medical Research, University of Wisconsin-Madison, Madison, WI 53706 USA; 2 Laboratory of Genetics & Wisconsin Institute for Discovery, University of Wisconsin-Madison, Madison, WI 53706 USA; 3 Morgridge Institute for Research, University of Wisconsin-Madison, Madison, WI 53706 USA; 4 Howard Hughes Medical Institute, University of Wisconsin-Madison, Madison, WI 53706 USA; 5 Present address: Developmental and Molecular Pathways, Novartis Institutes for Biomedical Research, 181 Massachusetts Avenue, Cambridge, MA 02139 USA

**Keywords:** Genome-wide profiling, Estrogen receptor α, RNA polymerase II, Ctr9, Breast cancer

## Abstract

**Background:**

We had previously identified Ctr9, the key scaffold subunit of the human RNA polymerase II (RNAPII) associated factor complex (PAFc), as a key factor regulating a massive ERα target gene expression and ERα-positive breast cancer growth. Furthermore, we have shown that knockdown of Ctr9 reduces ERα protein stability and decreases the occupancy of ERα and RNAPII at a few ERα-target loci. However, it remains to be determined whether Ctr9 controls ERα-target gene expression by regulating the global chromatin occupancy of ERα and RNAPII in the presence of estrogen.

**Results:**

In this study, we determined the genome-wide ERα and RNAPII occupancy in response to estrogen treatment and/or Ctr9 knockdown by performing chromatin immunoprecipitation coupled with high-throughput sequencing (ChIP-seq). We found that loss of Ctr9 dramatically decreases the global occupancy of ERα and RNAPII, highlighting the significance of Ctr9 in regulating estrogen signaling in ERα-positive breast cancer cells. Combining this resource with previously published genomic data sets, we identified a unique subset of ERα and Ctr9 target genes, and further delineated the independent function of Ctr9 from other subunits in PAFc when regulating transcription.

**Conclusions:**

Our data demonstrated that Ctr9, independent of other PAFc subunits, controls ERα-target gene expression by regulating global chromatin occupancies of ERα and RNAPII.

**Electronic supplementary material:**

The online version of this article (doi:10.1186/s12864-016-3248-3) contains supplementary material, which is available to authorized users.

## Background

Estrogen receptor α (ERα) is the paramount transcription factor in ERα-positive breast cancer, which constitutes 70% of all human breast cancers. Emerging evidence has shown that the execution of ERα signaling cascade is through multiple pathways to regulate the expression of ERα target genes in the presence or absence of ligand, i.e. 17β-estradiol (estrogen; E2) [1, 2]. The genomic ERα signaling pathway involves ligand binding, receptor dimerization and recognition of cognate estrogen response element (ERE) which consists of a consensus sequence containing the inverted repeat GGTCAxxxTGACC (where x is any nucleotide), located in either the distal or promoter regions of ERα-target genes [1]. The ERα-dependent transcriptional program is delicately regulated by a large number of cofactors including pioneering factors, chromatin modifiers as well as basal transcriptional machineries [3]. Perturbations of ERα-dependent transcriptional networks by the aberrant expression of ERα cofactors have been strongly linked to breast tumorigenesis [4]. Thus, comprehensively understanding their mechanisms of action could lead to therapeutic interventions for the treatment of ERα-positive breast cancer.

Human RNA polymerase II (RNAPII) associated factor complex (PAFc) is composed of five subunits including PAF1, Ctr9, Cdc73 (also known as parafibromin), Leo1, and eukaryotic specific Ski8 (also known as WDR61) [5]. PAFc is evolutionarily conserved from yeast to Drosophila and humans and it participates in the step-wise regulation of transcription from transcriptional initiation, elongation, to termination [6–8]. The PAFc regulated transcriptional elongation is coupled with histone modifications such as histone H2B mono-ubiquitination (H2Bub1), H3K4 tri-methylation (H3K4me3), and H3K36 tri-methylation (H3K36me3) via protein-protein interaction of PAFc subunits with the respective histone modifying enzymes [9]. Additionally, PAFc plays a role in mRNA processing and maturation, through the maintenance of proper poly (A) tail length [10]. Emerging evidence has demonstrated the diverse functions of human PAFc as well as its individual subunits in various cellular milieus. For instance, the human PAFc is required for the maintenance of embryonic stem cell identity [11], neuronal migration in mammalian brain [12], antiviral and pro-inflammatory response [13], and oncogenesis regulation [14, 15]. Our studies have demonstrated the involvement of Ctr9, a key scaffold subunit of human PAFc, in ERα-positive breast cancer progression and ERα target gene expression [16]. Specifically, using loss-of-function approach, we observed that depletion of Ctr9 led to apparent morphological change, decrease of proliferation, reduced colony formation, and impaired ERα-target gene expression in ERα-positive breast cancer cells [16]. Moreover, Ctr9 regulates ERα protein stability and facilitates RNAPII recruitment and transcription-coupled histone modifications such as H2Bub1, H3K27ac, and H3K36me3 at the selected ERα targets [16]. However, the global control of Ctr9 on ERα occupancy and RNAPII recruitment awaits investigation to deeply understand the functional significance of Ctr9 in ERα transcription network in breast cancer.

Profiling the genome-wide RNAPII and ERα chromatin occupancy affected by Ctr9 is advantageous over gene expression study to elucidate the global effects of Ctr9 on ERα-dependent transcriptional program and to identify the target genes regulated by Ctr9. In agreement with several published gene expression studies in MCF7 cells [17–28], our gene expression profiling identified less than 2000 E2-responsive genes [16, 29]. This is in contrast to the large scale ERα ChIP profiling where much more ERα binding sites across the genome were identified [18, 19, 21, 23, 25, 30–32]. For example, the Myles Brown lab identified 3665 ERα binding sites [25] and the Hendrik G Stunnenberg lab identified 10,205 ERα binding sites [32] in response to E2 treatment, by ChIP-chip and ChIP-seq, respectively. The discordance in E2-responsive transcription and ERα binding events is probably due to the prevalence of enhancers over promoters in regulating transcription and also suggests that some ERα binding events do not fulfill gene regulation. In addition, the mRNA level of a target gene may not faithfully reflect the transcriptional event due to the mRNA degradation. Therefore profiling the genome-wide RNAPII occupancy would provide a direct readout of transcriptional activity and thus yield mechanistic insights into the transcriptional regulation by estrogen and Ctr9 in ERα-positive breast cancer.

In this study, we performed chromatin immunoprecipitation followed by massive parallel sequencing (ChIP-seq) and demonstrated that Ctr9 knockdown (KD) elicits global decrease of the genome-wide occupancy of ERα and RNAPII, consistent with the function of Ctr9 in regulating ERα protein stability and ERα target gene expression. Integrative analyses of the ChIP-seq and microarray gene expression data sets identified the primary ERα and Ctr9 target genes, which have prognostic value of breast cancer outcome. Comparative analysis of RNAPII genome-wide occupancy alteration resulted from Ctr9 KD and PAF1 KD provided the first line of evidence in support of the PAFc independent function of Ctr9 on transcription regulation.

## Results

### Ctr9 knockdown triggers genome-wide decrease of ERα occupancy

**Fig. 1:**
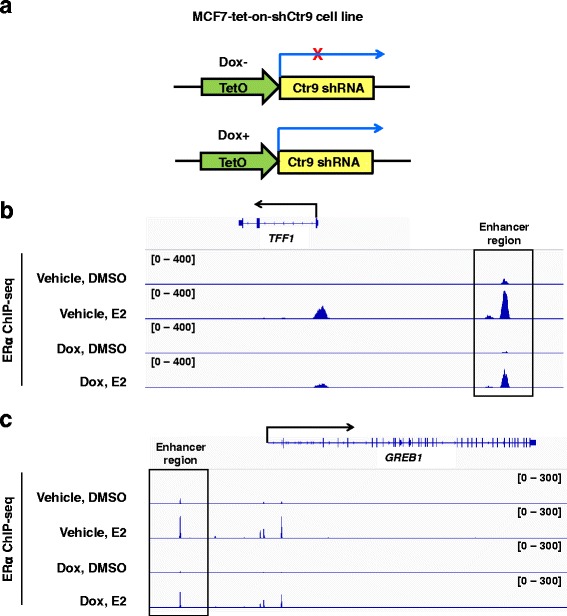
ERα occupancy at selected ERα target genes. **a** Schematic representation of the design of doxycycline (Dox) inducible Ctr9 knockdown in MCF7 cells. **b** Representative genome browser views of ERα ChIP-seq profiles at *TFF1* gene under the four indicated treatment conditions. **c** Representative genome browser views of ERα ChIP-seq profiles at *GREB1* gene under the four indicated treatment conditions

To investigate the estrogen and Ctr9 mediated transcription regulation in ERα-positive breast cancer cells, we used the MCF7-tet-on-shCtr9 inducible knockdown cell line previously developed in our lab as a model system (Fig. 1a) [16, 33]. ChIP-seq was first performed using an anti-ERα antibody in MCF7-tet-on-shCtr9 cells pre-treated with vehicle or doxycycline (Dox) followed by DMSO control or E2 induction. Classical ERα target genes, such as *TFF1* and *GREB1*, showed significant enrichment of ERα over a narrow range in their enhancer and promoter regions in response to E2 treatment (Fig. 1b and c), These results are highly consistent with several previous reports [18, 25, 32]. Consistent with our previous ChIP-qPCR results [16], we found that Ctr9 KD by Dox treatment significantly reduced ERα binding at the enhancer and promoter regions of *TFF1* and *GREB1* genes (Fig. 1b and c).

**Fig. 2:**
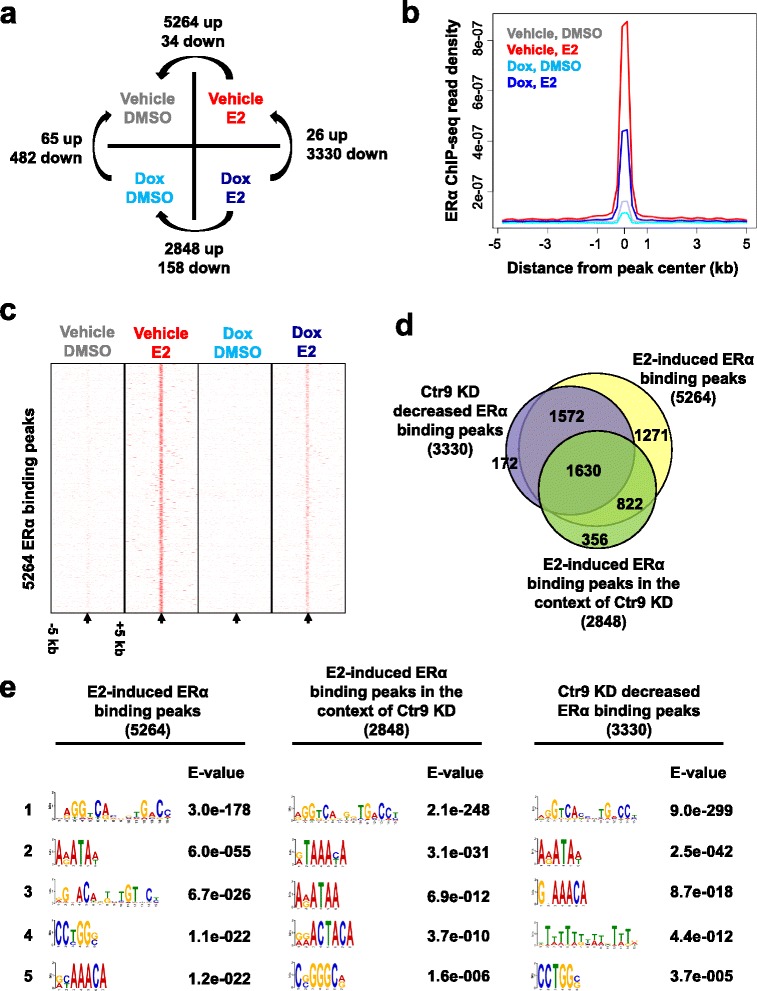
Ctr9 knockdown decreases genome-wide ERα occupancy. **a** A matrix describing the approach used to identify differential ERα binding sites. **b** Aggregate plot showing the genomic binding profiles of ERα on ERα binding regions under the indicated four treatment conditions. **c** Heatmaps showing the ERα occupancy on ± 5 kb of ERα binding peak centers in the indicated four treatment conditions. The *black arrow* denotes the center of ERα binding peaks. **d** Venn diagram showing the overlaps between E2-induced ERα binding sites, E2-induced ERα binding sites in the context of Ctr9 knockdown (KD), and Ctr9 KD decreased ERα binding sites in the presence of E2. **e** De novo motif analyses of the three sets of ERα binding sites noted in (**d**)

In accordance with others’ findings that substantial ERα binding is induced by estrogen treatment [25, 32], we identified a total of 5264 significant ERα binding sites upon E2 induction (Fig. 2a). We visually inspected the genomic locations of these 5264 ERα binding sites using Cistrome [34], and found that the majority of the binding sites are located within introns (42.1%) or distal regions (44.4%) and only a small percentage (7.0%) in promoter regions (Additional file 1: Figure S1) in agreement with previous reports [23, 25, 32], validating the reliability of our ChIP-seq datasets. Comparison between the four treatment conditions revealed that, in the presence of E2, Ctr9 KD decreased ERα binding at 3330 sites and only increased ERα binding at 26 sites (Fig. 2a), suggesting that Ctr9 is in general a positive regulator of genome-wide ERα occupancy. Aggregate plots further confirmed that loss of Ctr9 dramatically decreased the genome-wide ERα binding (Fig. 2b). As a further support, the heatmaps of ERα ChIP-seq density showed that Ctr9 depletion significantly reduced ERα occupancy at the majority of ERα binding sites (Fig. 2c). To obtain a more intuitive view of the effect of Ctr9 KD on global ERα binding, we performed a Venn diagram analysis for 5264 E2-induced ERα binding sites in control cells, 2848 E2-induced ERα binding sites in Ctr9 KD cells, and 3330 ERα binding sites decreased by Ctr9 KD (Fig. 2d). Among the E2-induced ERα binding sites in control cells, 1630 was significantly reduced by Ctr9 KD, although E2 treatment can still induce the binding of ERα at these sites (Fig. 2d). Much stronger effect was observed for 1572 ERα binding sites where Ctr9 KD almost completely abrogated the ERα binding (Fig. 2d). Therefore, we termed the 1630 sites and 1572 sites less and more sensitive to Ctr9 KD, respectively. ERE motif analyses identified that 65 and 55% of these two groups of ERα cistrome contains at least half ERE, respectively. We had previously shown that Ctr9 KD decreased ERα protein level thus expected reduced ERα occupancy on all targets. These results instead support the notion that Ctr9 elicits target-specific regulation towards ERα target genes, underscoring the complexity of Ctr9 function in the regulation of ERα-mediated transcriptional program beyond its conventional roles in the PAFc to regulate transcription initiation and elongation.

### Ctr9 knockdown alters the repertoire of ERα cistrome as illustrated by motif analyses

Next, we further investigated the impact of Ctr9 KD on ERα cistrome. We interrogated the sequence of the aforementioned three groups of ERα binding sites (Fig. 2d) for overrepresentation of DNA motifs using the MEME suite [35]. As expected, the most prevalent motifs at the ERα binding sites in all three groups are EREs (Fig. 2e, No. 1). Moreover, among the top five DNA motifs for E2-induced ERα cistrome, we found significant enrichment of DNA sequences featuring progesterone responsive element (PRE) (Fig. 2e, No. 3) and FOXA1 binding motif (Fig. 2e, No. 5). These results are consistent with the previous reports that progesterone receptor associates with ERα and modulates ERα action in breast cancer [31], and FOXA1 is the pioneer factor for shaping the genome-wide distribution of ERα [36], respectively. Importantly, PREs are no longer enriched in the E2-induced ERα cistrome in the context of Ctr9 KD, this is consistent with our previous finding that Ctr9 KD significantly decreases the expression of progesterone receptor [16]. Furthermore, all three groups of ERα cistrome displayed overlapping yet different DNA motif enrichment, suggesting that Ctr9 might affect or cooperate with other factors to regulate ERα action and estrogen signaling in ERα-positive breast cancer.

### Ctr9 positively regulates RNA polymerase II binding in MCF7 cells

**Fig. 3:**
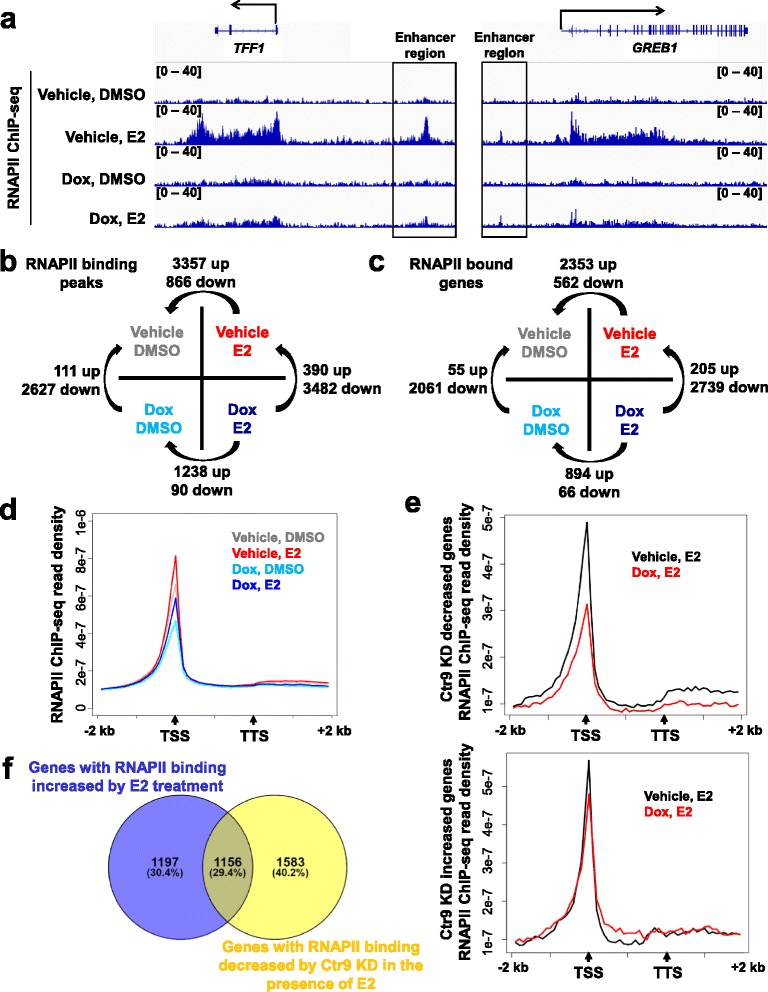
Ctr9 knockdown decreases genome-wide RNA polymerase II occupancy. **a** Representative genome browser views of RNA polymerase II (RNAPII) ChIP-seq profiles at *TFF1* and *GREB1* genes under the four indicated treatments. **b** A matrix describing the approach used to identify differential RNAPII binding peaks. **c** A matrix describing the approach used to identify genes with differential RNAPII binding peaks. RNAPII-bound gene is defined as gene that contains one or more RNAPII binding peaks within the gene body. **d** Metagene analysis showing total RNAPII occupancy measured by ChIP-seq in cells under the four indicated treatments. All RNAPII-bound genes were normalized to same length with 2 kb extended upstream from TSS and 2 kb downstream from TTS for analyzing the average density. TSS, transcription start site; TTS, transcription termination sites. **e** Metagene analyses showing total RNAPII occupancy measured by ChIP-seq for Ctr9 knockdown decreased genes (upper) and increased genes (bottom) selected from the microarray gene expression data set. **f** Venn diagram showing the overlap between genes with RNAPII binding increased by E2 treatment and genes with RNAPII binding decreased by Ctr9 KD in the presence of E2

Given that the vast majority of ERα binding sites are located at great distances from the annotated genes (Additional file 1: Figure S1), identification of ERα direct target genes simply based on the ERα binding sites has been problematic. To identify genes in response to E2 and/or Ctr9 KD in a more intuitive manner, we performed ChIP-seq for RNAPII in MCF7-tet-on-shCtr9 cells under four different conditions (Vehicle, DMSO; Vehicle, E2; Dox, DMSO; Dox, E2). RNAPII occupancy throughout the gene body represents a direct readout of the transcriptional activity and thus provides more intuitional evidence for estrogen and Ctr9 mediated transcription regulation. Classical ERα target genes, for example *TFF1* and *GREB1*, showed a dramatic increase of RNAPII occupancy over their enhancer, promoter, and gene body regions upon E2 treatment (Fig. 3a). Ctr9 KD by Dox treatment significantly reduced RNAPII occupancy at the aforementioned regions of *TFF1* and *GREB1* genes (Fig. 3a), in line with our previous ChIP-qPCR results and microarray gene expression results [16]. Genomic location analysis of E2-induced RNAPII binding sites showed that unlike ERα, RNAPII binding displayed more dispersed occupancy across the genome (Additional file 1: Figure S2).

At a global scale, E2 treatment and Ctr9 KD triggered redistribution of genome-wide RNAPII binding pattern (Fig. 3b). To directly identify genes responding to E2 treatment and/or Ctr9 KD, we first defined RNAPII-target gene using the criteria that one or more RNAPII binding sites can be found within its gene body (TSS, TTS, 5′-UTR, exon, intron, and 3′-UTR). Comparisons between the different conditions showed that, in response to E2 treatment, 2353 genes showed an increase and 562 genes showed a decrease in RNAPII occupancy (Fig. 3c). In contrast, E2 stimulation only increased RNAPII occupancy over 894 genes in Ctr9 KD cells (Fig. 3c), suggesting that Ctr9 positively regulates E2-induced RNAPII binding. The positive regulatory role of Ctr9 was further supported by the decrease of RNAPII occupancy over thousands of genes upon Ctr9 KD (Fig. 3c). Aggregate plot also confirmed that loss of Ctr9 significantly decreased the genome-wide RNAPII occupancy particularly at the transcription start sites (TSS) (Fig. 3d). We previously performed microarray gene expression analysis in MCF7-tet-on-shCtr9 cells treated with vehicle or Dox followed by DMSO control or E2 induction to identify E2 and Ctr9 regulated transcriptome [16, 29]. To gain additional insights into the regulation of RNAPII occupancy by Ctr9, we further examined the RNAPII occupancy for Ctr9 KD decreased and increased genes selected from our microarray data set (Fig. 3e). Aggregate plots showed that RNAPII occupancy was drastically decreased by Ctr9 KD for Ctr9 KD decreased genes, while increased particularly within the gene body by Ctr9 KD for Ctr9 KD increased genes, suggesting the context dependent role of Ctr9 in regulating RNAPII occupancy in MCF7 cells. To directly assess the impact of Ctr9 KD on estrogen signaling, we compared the genes with RNAPII binding increased by E2 treatment and genes with RNAPII binding decreased by Ctr9 KD in the presence of E2 (Fig. 3f). The Venn diagram analysis showed that about half of E2-responsive genes (1156 genes) displayed RNAPII binding decrease upon Ctr9 KD (Fig. 3f). Besides, Ctr9 KD also decreased RNAPII occupancy at 1583 non-E2-responsive genes (Fig. 3f), suggesting that Ctr9-dependent RNAPII chromatin occupancy is not restricted to the estrogen regulated genes in breast cancer.

### Integrative analyses of the combined ChIP-seq and microarray data reveal the ERα and Ctr9 signature which has clinical significance

**Fig. 4:**
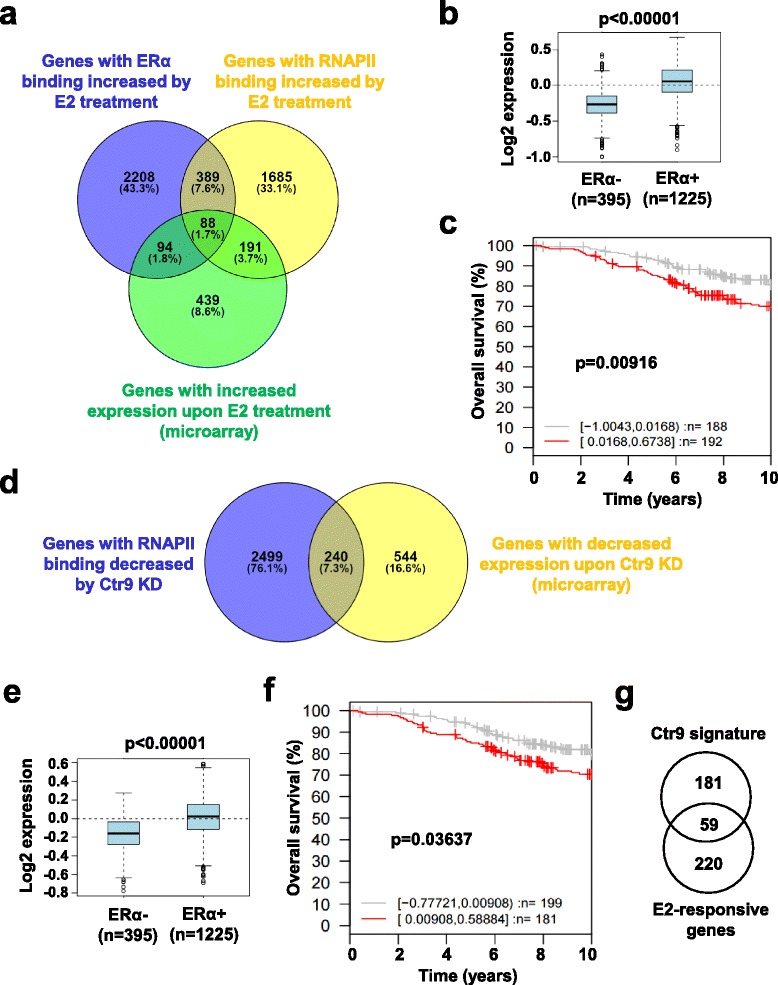
Integrative analyses of ChIP-seq and microarray gene expression data sets identify primary ERα and Ctr9 targets. **a** Venn diagram showing the overlaps between genes with ERα binding increased by E2 treatment, genes with RNAPII binding increased by E2 treatment, and genes with increased expression upon E2 treatment identified from microarray analysis. **b** Box plots displaying the mRNA expression of primary ERα target gene signature between ERα- (*n* = 395) and ERα + (*n* = 1225) breast tumors. The gene expression data were accessed using the Gene Expression-Based Outcome for Breast Cancer Online (GOBO) tool. **c** Kaplan-Meier survival analysis displaying the relationship between overall survival time and the primary ERα target gene signature in ERα+, lymph node negative (LN-) breast cancer patients. The breast cancer outcome-linked gene expression data were accessed using the GOBO tool. **d** Venn diagram showing the overlap between genes with RNAPII binding decreased by Ctr9 knockdown and genes with decreased expression upon Ctr9 knockdown identified from microarray analysis in the presence of E2. **e** Box plots displaying the mRNA expression of Ctr9 target gene signature between ERα- (*n* = 395) and ERα + (*n* = 1225) breast tumors. The gene expression data were accessed using the GOBO tool. **f** Kaplan-Meier survival analysis displaying the relationship between overall survival time and the Ctr9 target gene signature in ERα+, LN- breast cancer patients. The breast cancer outcome-linked gene expression data were accessed using the GOBO tool. **g** Venn diagram showing the overlap between the 240 Ctr9 target genes noted in (**d**) and the 279 E2-activated genes noted in (**a**)

To directly identify ERα and Ctr9 targets, we performed integrative analyses of the combination of ChIP-seq and microarray data. Comparing genes with RNAPII binding increased by E2 and genes with increased expression upon E2 treatment revealed an overlap of 279 genes, termed E2-activated genes (Fig. 4a and Additional file 2: Table S1). Further comparing with genes with ERα binding increased by E2 treatment identified 88 common genes among the three groups including some well-known ERα target genes such as *TFF1* and *GREB1* (Fig. 4a and Additional file 2: Table S1). Thus, we defined the 88-gene set as primary ERα target signature. Note that many genes with increased expression upon E2 treatment failed to display elevated RNAPII binding (Fig. 4a), probably because ChIP-seq with short E2 treatment (45 min) predominantly identifies early responsive target genes whereas gene expression profiling at 4 h after E2 treatment identifies the late responsive and some indirect targets. As ERα signature, the identified 88 gene set is highly expressed in ERα-positive human breast tumors (Fig. 4b) and the high expression level of the ERα signature is associated with poor overall survival in ERα-positive, lymph node-negative breast cancer patients (Fig. 4c).

Given that ERα-positive breast cancer depends on estrogen for growth and target gene transcription regulation, we next sought to identify primary Ctr9 targets in the presence of E2 by comparing genes with RNAPII binding decreased by Ctr9 KD and genes with decreased expression upon Ctr9 KD (Fig. 4d). The Venn diagram analysis identified 240 common genes between these two data sets. We defined the 240 gene set as Ctr9 signature (Fig. 4d and Additional file 2: Table S1). To determine if the Ctr9 signature has a broader clinical significance, we mined breast cancer outcome-linked gene expression data using the Gene Expression-Based Outcome for Breast Cancer Online (GOBO) tool [37]. In line with our previous finding that Ctr9 is highly expressed in ERα-positive breast cancer [16], the Ctr9 signature is also enriched in ERα-positive breast tumors (Fig. 4e). Kaplan-Meier analysis demonstrated that high expression levels of the Ctr9 signature strongly associates with poor overall survival in ERα-positive, lymph node-negative breast cancer patients (Fig. 4f), further highlighting the functional significance of Ctr9 in human breast cancer. Furthermore, comparing the Ctr9 signature with E2-responsive genes revealed an overlap of 59 genes (Fig. 4g and Additional file 2: Table S1). Taken together, these integrative analyses provided a deeper understanding of estrogen and Ctr9 regulated transcriptome in ERα-positive breast cancer cells.

### Distinct roles of Ctr9 in regulating RNAPII occupancy independent of PAFc

**Fig. 5:**
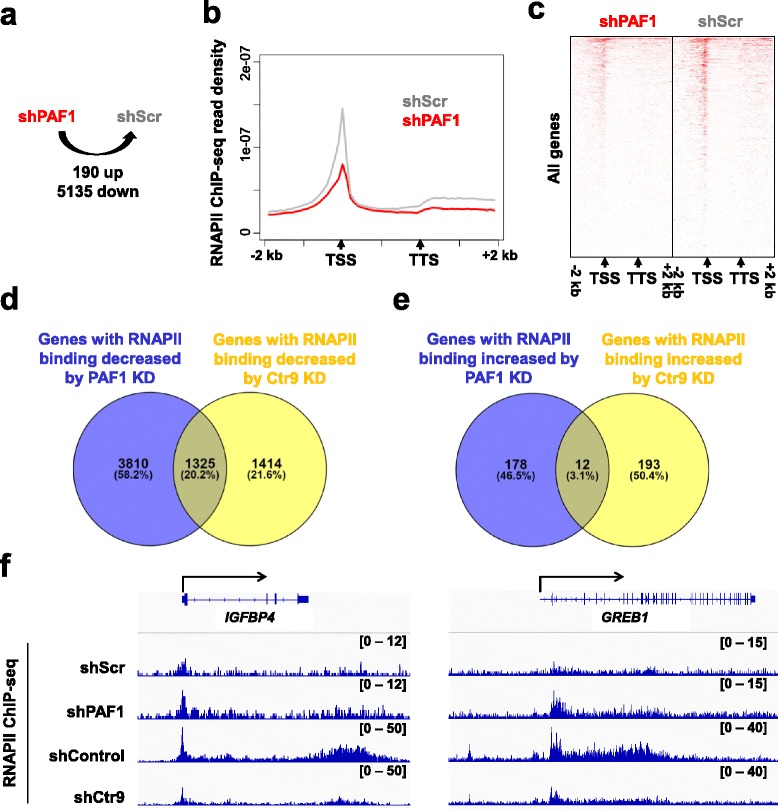
Overlapped and distinct roles of PAF1 and Ctr9 in RNA polymerase II chromatin association. **a** Schematic representation of the number of genes with differential RNAPII binding in PAF1 knockdown MCF7 cells (shPAF1) as compared to control cells (shScr). **b** Metagene analysis showing total RNAPII occupancy measured by ChIP-seq in cells transduced with shScr (control) or shPAF1. All RNAPII-bound genes were normalized to same length with 2 kb extended upstream from TSS and 2 kb downstream from TTS for analyzing average density. TSS, transcription start site; TTS, transcription terminal sites. **c** Heatmap showing RNAPII occupancy at all genes in PAF1 knockdown (shPAF1) and control (shScr) conditions. **d** Venn diagram showing the overlap between genes with RNAPII binding decreased by PAF1 knockdown and genes with RNAPII binding decreased by Ctr9 knockdown. **e** Venn diagram showing the overlap between genes with RNAPII binding increased by PAF1 knockdown and genes with RNAPII binding increased by Ctr9 knockdown. **f** Representative genome browser views of RNAPII ChIP-seq profiles at *IGFBP4* and *GREB1* genes in the indicated conditions

**Fig. 6:**
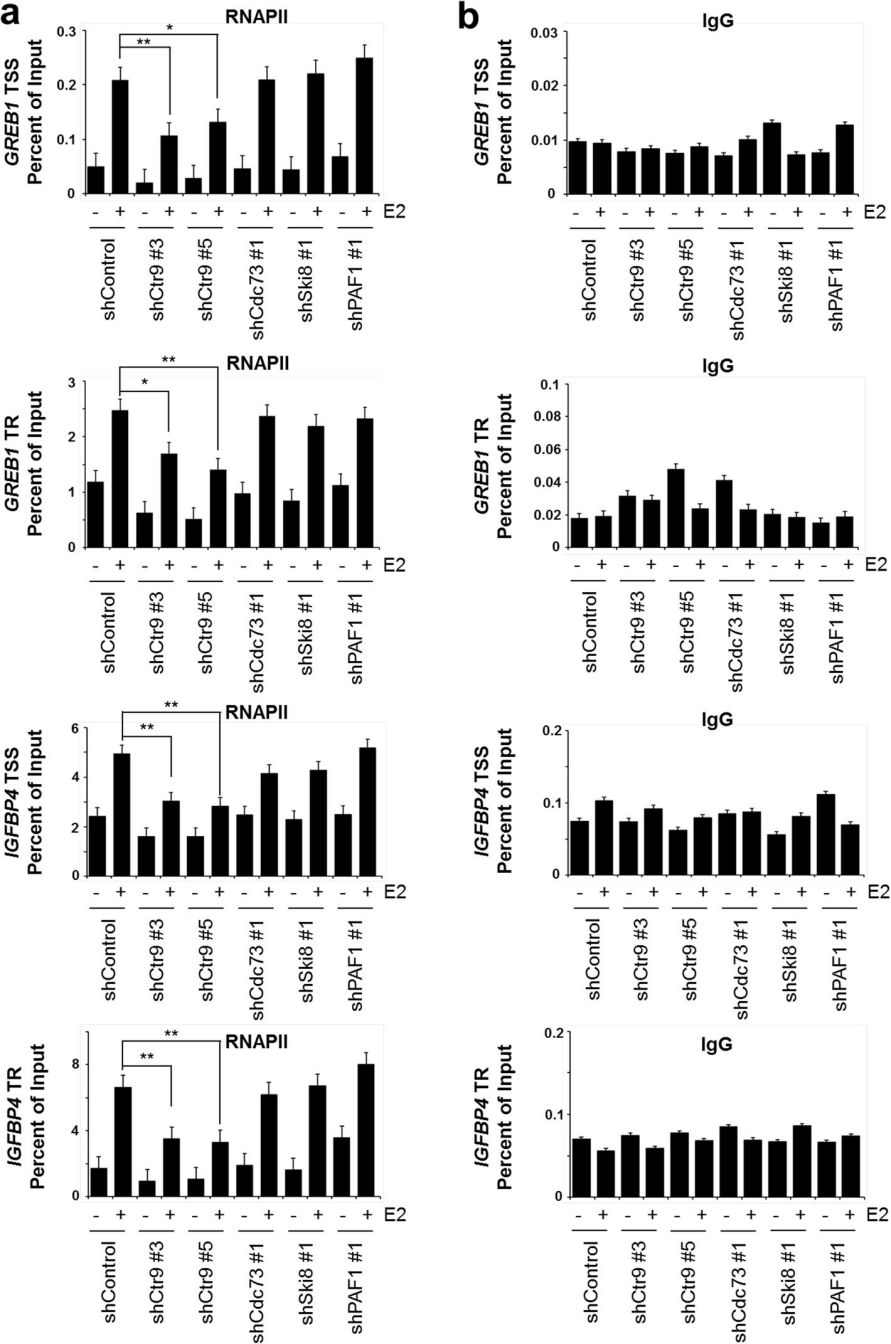
PAFc-independent role of Ctr9 in RNA polymerase II chromatin association at GREB1 and IGFBP4 genes assessed by ChIP-qPCR. **a** ChIP-qPCR analyses of the occupancy of RNAPII on the TSS or TR of *GREB1* and *IGFBP4* genes in MCF7 cells stably expressing control shRNA or shRNAs targeting Ctr9, Cdc73, Ski8, and PAF1 treated with DMSO or 10 nM E2 for 45 min. **b** ChIP-qPCR analyses of the occupancy of IgG control on the TSS or TR of *GREB1* and *IGFBP4* genes in MCF7 cells stably expressing control shRNA or shRNAs targeting Ctr9, Cdc73, Ski8, and PAF1 treated with DMSO or 10 nM E2 for 45 min. ChIP-qPCR data were normalized to input DNA qPCR data and expressed as “percent of input”; data are represented as mean ± SD. *n* = 3

Our previous study implies that Ctr9 might function independently of the other subunits of PAFc in regulating target gene expression and breast cancer progression [16]. To further distinguish the functions of Ctr9 from the other PAFc subunits, we analyzed recently published RNAPII ChIP-seq data obtained in wild-type and PAF1 KD MCF7 cells cultured in complete medium [38]. Similar to Ctr9 KD, PAF1 KD resulted in decrease of RNAPII occupancy over 5135 genes and increase over only 190 genes (Fig. 5a), suggesting that PAF1 is a positive regulator of RNAPII genomic binding in MCF7 cells. Aggregate plot and heatmap of ChIP-seq density also confirmed that loss of PAF1 significantly decreases genome-wide RNAPII occupancy (Fig. 5b and c). To examine whether PAF1 and Ctr9 exhibit the same regulation on genome-wide RNAPII occupancy, we compared the genes that elicited decreased RNAPII binding by knocking down PAF1 and Ctr9; the results showed an overlap of 1325 genes (Fig. 5d). Interestingly, a large number of PAF1 regulated genes and Ctr9 regulated genes are mutually exclusive (Fig. 5d), indicating that Ctr9 functions independently from PAF1. Similarly, comparison of genes that elicited increased RNAPII binding by knocking down PAF1 and Ctr9 revealed only 12 common genes (Fig. 5e). Taken the Ctr9 target gene *IGFBP4* and *GREB1* as examples, Ctr9 KD dramatically decreased the occupancy of RNAPII across the gene body whereas PAF1 KD elicited no effect on RNAPII occupancy at *IGFBP4* gene and increased RNAPII occupancy at *GREB1* gene, respectively (Fig. 5f). To further validate our ChIP-seq findings, we performed validation experiments by ChIP-qPCR to examine the RNAPII occupancy at the transcription start site (TSS) and transcribed region (TR) of *GREB1* and *IGFBP4* genes in MCF7 cells stably expressing control shRNA or shRNAs targeting Ctr9, Cdc73, Ski8, and PAF1 (Fig. 6). The results showed that Ctr9 KD with two independent shRNAs (shCtr9 #3 and shCtr9 #5) significantly reduced the RNAPII occupancy at TSS and TR regions of *GREB1* and *IGFBP4* genes, whereas KD of the other PAFc subunits elicited little effect on the RNAPII occupancy at the same regions (Fig. 6). Altogether, these findings suggest that Ctr9 may have a unique role in regulating RNAPII chromatin association independent of PAFc.

## Discussion

Estrogen signaling in ERα-positive breast cancer is tightly regulated by ERα itself as well as its associated co-regulator proteins. It is essential to understand the ERα target gene network and the transcription regulation mechanisms. In this study, we have applied ChIP-seq to determine the genome-wide alterations of ERα and RNAPII occupancy in response to E2 induction and/or KD of Ctr9. The major finding is that loss of Ctr9 led to dramatic decrease of genome-wide occupancy of both ERα and RNAPII, supporting a positive regulatory role of Ctr9 in mediating estrogen-dependent transcriptional regulation. Integrative analyses of the combined ChIP-seq and microarray gene expression data led to the identification of 88 primary ERα targets and 240 primary Ctr9 targets which both have significant clinical relevance. We acknowledge that ChIP-seq for Ctr9 in MCF7 cells could provide a more precise identification of direct Ctr9 target genes. However, Ctr9 ChIP-seq has been technically challenging. We performed the Ctr9 ChIP-seq using the commercially available anti-Ctr9 antibody. However, ChIP-seq signals were very weak with less than 30 peaks identified (data not shown). This is consistent with the report by Fei Xavier Chen, et al. [38] in which this anti-Ctr9 antibody also failed in ChIP-seq. Therefore, ChIP-grade antibodies for Ctr9 need to be developed for future ChIP-seq experiments.

Emerging evidence has shown that some transcriptional cofactors can exert a global impact on ERα target gene expression and/or genome-wide ERα binding [18, 36, 39–41]. For example, loss-of-function approach coupled with gene expression microarray profiling demonstrated that BRD4, KDM3A, and Cohesin are required for the expression of a large portion of ERα target genes [18, 40–42]. GATA3 and FOXA1 have been implied as the pioneer factors for priming the genome-wide distribution of ERα [36, 39]. Although Ctr9 acts as a master regulator of estrogen signaling by stabilizing ERα protein [16], controlling genome-wide ERα and RNAPII binding, and affecting the target gene expression, the importance of Ctr9 in the estrogen signaling network orchestrated by the family of transcriptional factors and cofactors is of great interest and awaits future investigation. In this regard, our genomic analyses performed in this study provided valuable resources for possible comparison with the other factors regulating ERα transcriptome. Moreover, given that ERα protein level is regulated by Ctr9, we cannot distinguish whether the global change in ERα cistrome upon Ctr9 KD is specific to loss of Ctr9 or is due to reduced ERα protein itself, which awaits further clarification in the future.

Our previous study has also shown that Ctr9 modulates MCF7 cell morphology, cell proliferation, and ERα target gene expression [16]. However, we could not distinguish whether Ctr9 functions independently or in the context of PAFc. Here, by comparing the alterations in RNAPII occupancy resulted from PAF1 KD and Ctr9 KD, we provided the first evidence showing that PAF1 and Ctr9, despite both positively regulate genome-wide RNAPII binding, might regulate RNAPII binding through different mechanisms in MCF7 cells. Moreover, ChIP-seq analyses in human acute myeloid leukemia THP1 cells showed that PAF1 occupancy generally overlaps with the promoter-proximal RNAPII peaks whereas Ctr9 occupancy does not [43], reinforcing the PAFc independent functions of Ctr9.

## Conclusions

In this study, we profiled the genome-wide binding patterns of ERα and RNAPII in response to estrogen stimulation and/or Ctr9 KD. We showed that Ctr9 acts as a master regulator of the gene transcription program in ERα-positive breast cancer cells by affecting the global occupancy of ERα and RNAPII. Combination of ChIP-seq and the previous microarray gene expression data revealed primary target genes of ERα and Ctr9. Moreover, comparing RNAPII binding alterations between Ctr9 KD and PAF1 KD identified a unique subset of Ctr9 target genes that implicate PAFc-independent function of Ctr9. Altogether, our study provides a molecular basis to further understand the transcriptional control in ERα-positive breast cancers.

## Methods

### Cell culture

MCF7-tet-on-shCtr9 cells were generated previously [33] and maintained in Dulbecco’s modified medium (DMEM) (Life Technologies) supplemented with 10% fetal bovine serum (FBS) (Life Technologies). Cells were cultured at 37 °C in a humidified atmosphere containing 5% CO_2_. Doxycycline (Dox) was purchased from Clontech (Mountain View, CA) and used at a final concentration of 500 ng/ml. Dimethyl sulfoxide (DMSO) and 17 β-estradiol (E2) were purchased from Sigma (St. Louis, MO). Prior to chromatin immunoprecipitation (ChIP) assays, MCF7-tet-on-shCtr9 cells were cultured in DMEM supplemented with 10% FBS in the absence or presence of 500 ng/mL Dox for 4 days, followed by continuing cultured in phenol red-free DMEM supplemented with 5% charcoal-dextran-treated FBS in the absence or presence of 500 ng/mL Dox for another 3 days. Cells were then treated with DMSO or 10 nM E2 for 45 min. Antibodies used for ChIP-seq in this study were anti-ERα (sc-543, HC-20) purchased from Santa Cruz Biotechnology and anti-RNA polymerase II (#05-623, clone CTD4H8) purchased from Millipore.

### Chromatin immunoprecipitation (ChIP)

ChIP assays were performed as described previously [16]. In detail, cells were grown in 15-cm dish as described above and cross-linked for 10 min at room temperature by the addition of 37% formaldehyde stock solution to the cell culture media to achieve a final concentration of 1%. Crosslink was quenched for 5 min at room temperature by the addition of glycine to a final concentration of 0.125 M followed by two washes with ice-cold PBS. Cells were scraped, collected by centrifugation and subjected to cell lysis in lysis buffer 1 (10 mM HEPES pH 7.0, 10 mM EDTA, 0.5 mM EGTA, 0.25% Triton X-100, supplemented with 0.5 mM PMSF before use) with rotation for 10 min at 4 °C. The crude nuclear pellets were collected by centrifugation (1500 rpm, 4 °C, 4 min), washed with lysis buffer 2 (10 mM HEPES pH 7.0, 200 mM NaCl, 1 mM EDTA, 0.5 mM EGTA, supplemented with 0.5 mM PMSF before use) with rotation for 10 min at 4 °C. Nuclear pellets were collected by centrifugation (1500 rpm, 4 °C, 4 min), resuspended in nuclear lysis buffer [50 mM Tris–HCl pH 8.1, 10 mM EDTA, 1% SDS, supplemented with 1 mM PMSF and 1 × protease inhibitor cocktail (Sigma-Aldrich) before use] and incubated on ice for 10 min. The chromatin was sheared by sonication in ice-water bath at 4 °C using a Branson Sonifier 450 with a microtip (40% amplitude, 3 s on, 10 s off, 5 min total pulse time). Sonicated nuclear lysates were cleared by centrifugation (15,000 rpm, 10 °C, 15 min) and nuclear protein concentration was determined using the BioRad Protein Assay (BioRad). Equal amounts of total nuclear proteins were used for ChIP and supplemented with corresponding volume of nuclear lysis buffer to achieve same final volumes between different samples, and then diluted 1:10 with dilution buffer (20 mM Tris–HCl pH 8.1, 150 mM NaCl, 2 mM EDTA, 1% Triton X-100, supplemented with 1 × protease inhibitor cocktail before use). Five percent of the material was removed and saved as input, and the rest was incubated overnight at 4 °C with the antibody of interest or a normal IgG control.

The following day, the immune complexes were collected by adding Dynabeads Protein A or Dynabeads M-280 Sheep anti-Mouse IgG (Life Technologies) (beads were pre-washed with ChIP dilution buffer three times before use) and incubating with rotation for 2 h at 4 °C. The immunoprecipitated material was subsequently washed once with low salt wash buffer (20 mM Tris–HCl pH 8.1, 150 mM NaCl, 2 mM EDTA, 0.1% SDS, 1% Triton X-100), once with high salt wash buffer (20 mM Tris–HCl pH 8.1, 500 mM NaCl, 2 mM EDTA, 0.1% SDS, 1% Triton X-100), once with LiCl wash buffer (10 mM Tris–HCl pH 8.1, 0.25 M LiCl, 1 mM EDTA, 1% NP-40, 1% deoxycholate), and twice with TE buffer (10 mM Tris–HCl pH 8.0, 1 mM EDTA pH 8.0). Each wash was done with rotation for 5 min at 4 °C followed by separation on magnetic stand. The immunoprecipitated material was eluted twice at room temperature in freshly prepared elution buffer (1% SDS, 0.1 M NaHCO_3_) by shaking on a vortexer for 20 min. The eluted material and the input material were then digested with proteinase K (200 μg/ml final concentration) for 2 h at 55 °C, and the crosslinks were reversed overnight by incubating at 65 °C in a hybridization oven. DNA was purified using a Qiagen PCR Purification Kit according to the manufacturer’s protocol.

### ChIP-seq library preparation

Prior to ChIP-seq library preparation, ChIPed DNA samples were submitted to the Sequencing Facility at University of Wisconsin Madison Biotechnology Center for determining DNA concentration and size distribution, using Qubit Fluorometer (Thermo Fisher Scientific) and Agilent High Sensitivity DNA Kit (Agilent Technologies), respectively. 10 ng of ChIPed DNA from each condition was used to generate ChIP-seq library using the Ovation Ultralow System V2 1–16 Kit (NuGEN Technologies), according to the manufacturer’s protocol. Briefly, the DNA was end-repaired and subsequently ligated to Illumina sequencing adaptors. The ligated DNA was purified using the Agencourt RNAClean XP bead (Beckman Coulter). A subsequent PCR amplification step (15 cycles) added additional linker sequence to the purified fragments to prepare them for annealing to the Genome Analyzer flow-cell. Following PCR amplification, the library was size selected to a narrow range of fragment sizes by separation on a 2% agarose gel (120 V, 1.5 h) and a band between 300–500 bp was excised. The library was purified from the excised agarose using the Qiagen MiniElute PCR Purification Kit, according to the manufacturer’s protocol. Purified libraries were then submitted to the Sequencing Facility at University of Wisconsin Madison Biotechnology Center for quality control to determine the size, purity, and concentration of the final ChIP-seq libraries. Qualified libraries were submitted to New York Genome Center (NYGC) for deep sequencing using an Illumina HiSeq 2000 per the manufacturer’s instructions.

### ChIP-seq data analyses

ChIP-seq raw data were obtained from NYGC, and the reads were mapped to the human reference genome (UCSC hg19) using Bowtie (version 2.1.0) [44] using default parameters. ChIP-seq raw peaks were visualized in Integrative Genomics Viewer (IGV) (version 2.3.40) [45]. Peak calling was done by Model-based Analysis of ChIP-seq (MACS) (version 1.4.2) [46] using default parameters and by taking one treatment group as control and comparing the other treatment group to the corresponding control. Coverage was determined by normalizing the total number of mapped reads per hundred million. MEME suite (version 4.10.1) was used for motif discovery. The ±100 bp around a peak summit was used for motif discovery. Annotated genes with its 50 kb upstream that overlapped with ERα peaks were defined as ERα target genes. Annotated genes with their gene body overlapped with RNAPII peaks were defined as RNAPII target genes. For plots of ChIP reads over gene bodies, each gene was divided into 20 intervals (5% each interval) separately for the body of the genes, 2 kb upstream of the TSS, and 2 kb downstream of the TTS. Normalized ChIP read density for each interval was plotted. Standardized z-scores were used in heatmap visualization.

### Primers used in ChIP-qPCR experiments

- GREB1 TSS F: 5′-GCCAAATGGAAGAAGGACAG-3′
- GREB1 TSS R: 5′-ACCACCTACCTCCAGTCACC -3′
- GREB1 TR F: 5′-CCCAAGCCTTCTCTGGGTTC-3′
- GREB1 TR R: 5′-AGCAGACGAGAAGTAGGGCT-3′
- IGFBP4 TSS F: 5′-GGTGTGGCCAGGGACCGGTATAAA-3′
- IGFBP4 TSS R: 5′- AAGTTAGCAGGCGTGCCGGA-3′
- IGFBP4 TR F: 5′-TTGGGGAGGATGAGGGAGTG-3′
- IGFBP4 TR R: 5′-CCTCAACGCCAAAGTCCCTCTA-3′

## Additional files

Additional file 1: Figure S1. Genomic location of E2-induced ERα binding sites. **Figure S2.** Genomic location of E2-induced RNAPII binding sites. (DOCX 922 kb)
Additional file 2: Table S1. Identification of ERα and Ctr9 targets by analyzing the ChIP-seq and microarray data sets. (XLSX 15 kb)

## Abbreviations

ChIP-seq: chromatin immunoprecipitation coupled with high-throughput sequencing
ER: estrogen receptor
ERE: estrogen response element
PAFc: RNAPII associated factor complex
RNAPII: RNA polymerase II
