# Supplementary material for: Systematic identification of Ctr9 regulome in ERα-positive breast cancer
Source: BMC Genomics. 2016 Nov 9;17:902. doi: 10.1186/s12864-016-3248-3 (PMC5103509; doi:10.1186/s12864-016-3248-3)
Supplement: Additional file 1: Figure S1. — Genomic location of E2-induced ERα binding sites. Figure S2. Genomic location of E2-induced RNAPII binding sites. (DOCX 922 kb) [file 12864_2016_3248_MOESM1_ESM.docx]

**Supplemental information**

**Supplemental Figure 1. Genomic location of E2-induced ERα binding sites**

Average percentage of ERα ChIP-seq enrichment signals over specific genomic features like promoters, 5’ UTR, 3’ UTR, coding exons, introns, and distal intergenic regions were analyzed using Cistrome. Both genome and ChIP-seq enrichment were compared. The majority of E2-induced ERα binding sites are located within introns (42.1%) or distal regions (44.4%).

**Supplemental Figure 2. Genomic location of E2-induced RNAPII binding sites**

Average percentage of RNAPII ChIP-seq enrichment signals over specific genomic features like promoters, 5’ UTR, 3’ UTR, coding exons, introns, and distal intergenic regions were analyzed using Cistrome. Both genome and ChIP-seq enrichment were compared.

**Supplemental Figure 1**


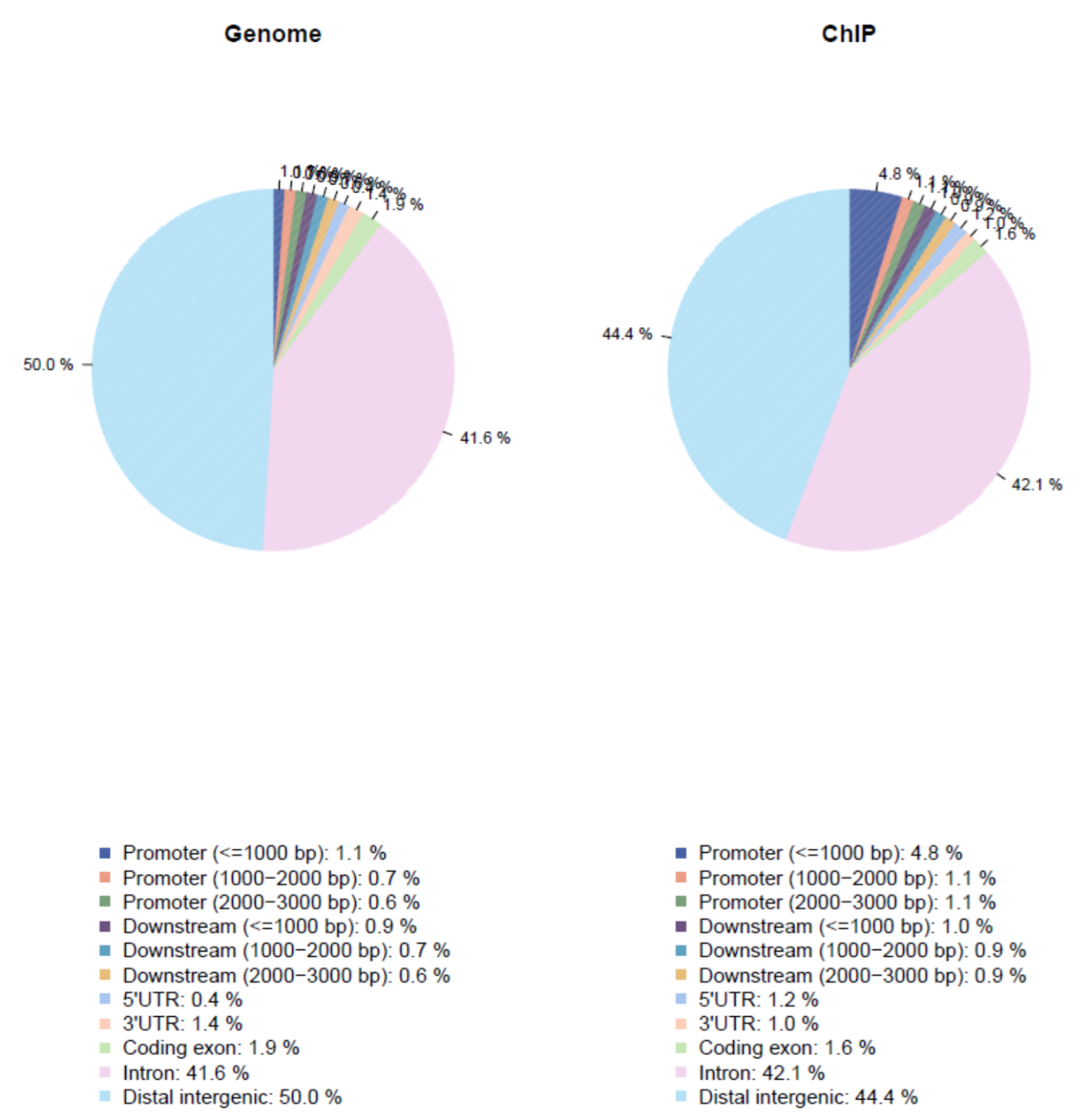


**Supplemental Figure 2**
